# Two-Minute Deep Learning–Powered Brain Quantitative Mapping: Accelerating Clinical Imaging With Synthetic Magnetic Resonance Imaging

**DOI:** 10.2196/79389

**Published:** 2026-01-23

**Authors:** Yawen Liu, Hongxia Yin, Zuofeng Zheng, Wenjuan Liu, Tingting Zhang, Linkun Cai, Haijun Niu, Han Lv, Zhenghan Yang, Zhenchang Wang, Pengling Ren

**Affiliations:** 1 Precision and Intelligence Medical Imaging Lab, Beijing Friendship Hospital, Capital Medical University, No.95 Yongan Road, Xicheng District, Beijing, 100050, China, 86 18810514627; 2 Department of Radiology, Beijing Friendship Hospital, Capital Medical University, Beijing, China; 3 Department of Medical Engineering, Beijing Friendship Hospital, Capital Medical University, Beijing, China; 4 Department of Radiology, Beijing Anzhen Hospital, Capital Medical University, Beijing, China; 5 Department of Radiology, Aerospace Center Hospital, Beijing, China; 6 School of Biological Science and Medical Engineering, Beihang University, Beijing, China

**Keywords:** magnetic resonance imaging, qMRI, fast MRI, deep learning, brain imaging

## Abstract

**Background:**

Quantitative magnetic resonance imaging (MRI) is an advanced technique that can map the physical properties (T1, T2, and proton density [PD]) of different tissues, offering crucial insights for disease diagnosis. Nonetheless, the practical application of this technology is indeed constrained by several factors, with the most notable being the protracted scanning duration.

**Objective:**

This study aimed to explore whether deep learning (DL)–based superresolution reconstruction of ultrafast whole brain synthetic MRI can obtain quantitative T1/T2/PD maps that are closely approximated to those from routine clinical scans, while substantially shortening scan time and preserving diagnostic image quality.

**Methods:**

A total of 151 healthy adults and 7 individuals with different pathologies were prospectively enrolled. Each individual was examined twice on a 3.0T scanner using routine and fast synthetic MRI protocols. The routine scans (acquisition matrix: 320×256) were interpolated to 512 by 512 for clinical display and served as reference images. The fast scans (acquisition matrix: 192×128) were preprocessed to 256 by 256 and used as inputs to a superresolution generative adversarial network (SRGAN), which reconstructed them to the same 512 by 512 interpolated resolution as the reference. For each quantitative chart, 120 (75.95%) healthy individuals’ images were used for training, and 38 (24.05%) individuals’ images (healthy individuals: n=31, 19.62%; patients: n=7, 4.43%) were used for testing. Agreement was assessed with a paired *t* test, two 1-sided tests, Bland-Altman analysis, and coefficients of variation.

**Results:**

DL reconstructed and reference T1/T2/PD values were strongly correlated (T1: *R*²=0.98; T2: *R*²=0.97; and PD: *R*²=0.99). The slopes of the linear regression were near 1.0 both for T1 (0.9418) and PD (0.9946), whereas T2 values were moderate, as the slope of the linear regression was 0.8057. Additionally, the average biases of T1, T2, and PD values were small (0.93%, −0.85%, and 0.31%, respectively). The intra- and intergroup coefficient of variation for most of the brain regions stayed below 5%, especially for PD values, and after DL reconstruction, it still has quantitative accuracy for lesions. Quantitative and qualitative analyses of image quality also indicate that SRGAN markedly suppressed noise and artifacts in fast acquisitions, restoring structural fidelity (structural similarity image measure) and signal fidelity (peak signal-to-noise ratio) close to the level of routine scans while substantially improving perceptual naturalness over fast scans (as measured by the naturalness image quality evaluator), although not yet matching that of routine imaging.

**Conclusions:**

SRGAN superresolution applied to ultrafast synthetic MRI yields whole brain T1, T2, and PD maps that show strong correlation with routine synthetic MRI while halving acquisition time and maintaining diagnostic image quality. Although T1 and PD values exhibit near-ideal agreement, and T2 values demonstrate a moderate systematic underestimation, this approach represents a promising step toward accelerating clinical deployment of quantitative brain imaging.

## Introduction

Quantitative magnetic resonance imaging (qMRI) can reflect the inherent properties of human tissue relaxation times and proton density (PD), providing valuable support for verifying visual assessments of structures and tissues against a normal quantitative standard, thereby holding significant clinical diagnostic value [1]. Nevertheless, the extended scan time of qMRI imposes limitations on its practical use. Synthetic MRI, based on multidynamic, multiecho sequences, can provide multiple quantitative information and multicontrast images in a single scan, enabling fast qMRI scans [2,3]. It has been proven to have comparable diagnostic value to conventional scanning methods, with the generated quantitative data being valuable for disease identification [4-7]. However, the single scan time of this imaging method still typically ranges from 4 to 7 minutes, particularly for brain MRI imaging, which can lead to individual discomfort and potentially impact image quality. Therefore, improving the imaging speed of synthetic MRI is the key to realizing ultrafast qMRI.

It is also crucial to ensure the quality of qMRI and the reliability of quantitative values while accelerating imaging. Usually, fast imaging methods such as parallel acquisition and compressed sensing may result in a loss of image quality [8]. The reconstruction technology based on deep learning (DL) has been applied in various medical image reconstruction tasks, and its development makes it possible to solve the trade-off between image quality and scan time. Generative adversarial networks, renowned for generating high-fidelity superresolution images, have been successfully used in MRI [9]. Many researchers have implemented the concept of generative adversarial networks for MRI image reconstruction, achieving high-quality reconstruction from highly undersampled data [10-14]. In addition, the generative adversarial approach can be used for tasks, such as artifact correction and denoising in MRI [15,16]. In clinical practice, in addition to focusing on image quality, it is more important to determine whether the DL-based reconstruction method can provide less bias and consistent quantitative data [17].

To shorten synthetic MRI quantitative acquisitions and increase clinical throughput, we hypothesized that DL superresolution can generate T1/T2/PD maps that are close to routine long acquisition quantitative values while maintaining image quality, thereby providing faster yet equally reliable neuroimaging and ultimately enhancing diagnostic precision and patient comfort.

## Methods

### Participants

This study was approved by the Medical Research Ethics Committees of the Beijing Friendship Hospital, Capital Medical University (2020-P2-122-02). Written informed consent was obtained from all participants prior to enrollment. A total of 151 healthy individuals were prospectively recruited at 1 institution for this study. The inclusion criteria were as follows: (1) the age of the individual was greater than or equal to 18 years, (2) the individual showed no structural changes or signs of disease in brain MRI, and (3) the individual showed no contraindications or adverse reactions to MRI examination. The exclusion criteria were as follows: (1) there were obvious artifacts in the images, and (2) the individual failed to complete all MRI examinations. In addition, we also collected images from 7 individuals with different pathologies (white matter [WM] hyperintensities, cerebral infarcts, and encephalomalacia) to evaluate the potential for clinical application.

### Synthetic MRI Acquisition

All MRI examinations were performed using a SIGNA Pioneer 3.0 T MRI scanner (GE Healthcare). All individuals underwent two synthetic MRI scans using the multidynamic multiecho sequence (MDME sequence, named MAGiC in the GE scanner): a routine scan protocol and a fast scan protocol. The routine scan uses commonly used clinical acquisition parameters: TR=4000 ms; TE1=18.3 ms; TE2=91.4 ms; TI=28.2 ms; thickness=5 mm; FOV=220×220 mm; acquisition matrix=320×256; echo-train length=16; bandwidth=31.25 kHz; delay times=170, 670, 1840, and 3840 ms; and acceleration factors=2, 24 slices, with a pixel size of 0.7×0.8 and an acquisition time of 4 minutes 55 seconds. Fast scan modifies the acquisition matrix and acceleration factor, which affect the acquisition time, by changing the acquisition matrix to 192×128 and the acceleration factor to 3. The remaining parameters remained the same as those of the routine scan, with a pixel size of 1.1×1.7 and an acquisition time of 1 minute 52 seconds.

### Data Preprocessing

The same method was used to retrieve the quantitative maps (T1 maps, T2 maps, and PD maps) as mentioned in a previous study [18]. In this study, quantitative maps from the routine scan (reconstruction matrix=512×512, 0.43×0.43 mm² pixel) served as the high-resolution (HR) reference, whereas those from the fast scan (reconstruction matrix=256×256, 0.86×0.86 mm² pixel) were used as low-resolution (LR) inputs. DICOM images were directly read and prepared for network training. It should be noted that the maximum image intensities of T1 maps, T2 maps, and PD maps are 43,000, 20,000, and 1600, respectively, which correspond to 10 times the actual values. This scaling is due to the rescaling slope value of 0.1 specified in the DICOM header file.

### Network Architecture

**Figure 1.**
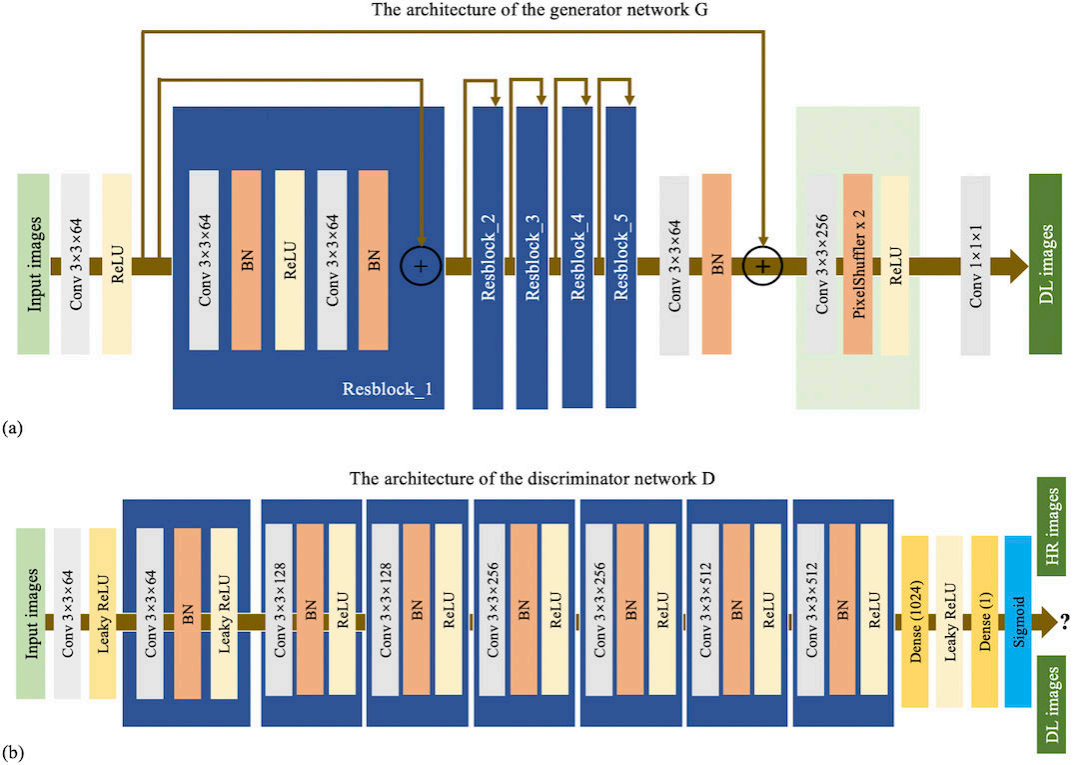
The architecture of the superresolution generative adversarial network. BN: batch normalization; DL: deep learning; HR: high resolution; ReLU: rectified linear unit.

The detailed generator network structure is shown in Figure 1A. It starts with a 9 by 9 convolutional filter and connects to 5 residual blocks, each of which consists of two 3 by 3 convolutional layers alternating with batch normalization layers, and activation is performed using a rectified linear unit (ReLU) function. After the residual network, a 3 by 3 convolution and a 1 by 1 convolution are connected, and a subpixel convolutional layer is used to upsample the image. In the generator network, except for the last layer that uses tanh as the activation function, all other layers use ReLU as the activation function. The discriminator network structure is shown in Figure 1B. In the discriminator network D, our model first uses an architecture comprising eight 3×3 convolutional layers and Leaky ReLU function activation. All convolutional layers except the first have batch normalization layers. The Sigmoid activation function is applied to the last fully connected layer, which outputs the probability of discriminating whether the input HR image is a real HR image or an image generated by the generator. The network architecture includes a pretrained VGG-19 network, which is used for feature extraction and loss function calculation.

### Loss Function

The pixel-by-pixel error method used is the L1 loss function, also known as the mean absolute error, which is calculated using equation 1. Although SRGAN completely discards the pixel-by-pixel error, we still add this error in a certain proportion during the actual training process to amplify the difference and guide the optimization of the model. The cross-entropy loss function of the discriminator is used as the adversarial error of the network, and the calculation method is expressed using equation 2. In addition to using the adversarial error, SRGAN also uses a content error, which is defined as the Euclidean distance between the superresolved image and the feature map of the reference image, and the calculation method is expressed using equation 3. The content error is used to align the content of LR images and HR images, which plays the same role as the mean square error. In this network, the pretrained VGG-19 network is used to extract the feature parameter map. Therefore, the loss function for the generator in this network is described using equation 4.


(1) $$L_{\text{mae}}=\frac{1}{CHW}\sum_{i,j,k}\left|I_{i,j,k}^{DL}-I_{i,j,k}^{GT}\right|$$

where $I_{DL}$is the output image of the generator and $I_{GT}$is the input reference image.


(2) $$L_{gan}=-\sum_{i}logD\left(G\left(I_{DL}\right)\right)$$

where G and D are the generative network and the discriminative network, respectively.


(3) $$L_{VGG}=\frac{1}{C_{j}H_{j}W_{j}}\sum_{x=1}^{W_{j}}\sum_{y=1}^{H_{j}}\sum_{c=1}^{C_{j}}{\left(\phi_{j}\left(G(I_{DL}{)}_{x,y,c}\right)-\phi_{j}\left(G(I_{GT}{)}_{x,y,c}\right)\right)}^{2}$$

where $\left(\emptyset_{j}\right)$ is the feature map obtained from the *j*th convolutional layer of the VGG-19 network, $C_{j}$, $H_{j}$, and $W_{j}$ are the number of channels and height and width of the feature map, respectively, $I_{DL}$ is the output image of the generator, and $I_{GT}$ is the input reference image.


(4) $$L_{total}=L_{mae}+10^{-3}L_{gan}+2\times 10^{-6}L_{VGG}$$

### Implementation

Approximately 80% (120 healthy individuals) of the dataset were allocated to training, and the remaining individuals (healthy individuals: 31, 19.62%; and patients: 7, 4.43%) were used for testing. Slices from each individual were pooled and randomly shuffled within their respective sets to prevent any sequential bias and improve the performance of the model. Before training, all quantitative maps (T1, T2, and PD) underwent intensity normalization to match the output range of the generator network, that is, $image_{norm}=\left(pixel_{value}/normalize_{para}\right)\times 2-1$, where normalize_para is set to 45,000, 22,000, and 1800 for T1, T2, and PD maps, respectively.

The DL models for T1, T2, and PD maps were trained separately but with identical hyperparameters. Each model was implemented using the TensorLayer framework [19] and optimized via the adaptive moment estimation (Adam) optimizer with a learning rate of 1×10^3^ and step-wise decay every 1000 iterations. Training was performed on an NVIDIA Tesla V100 GPU with a batch size of 8 and a total of 200 epochs. All other parameters were kept at their default settings.

### Quantitative Value Measurements

**Figure 2.**
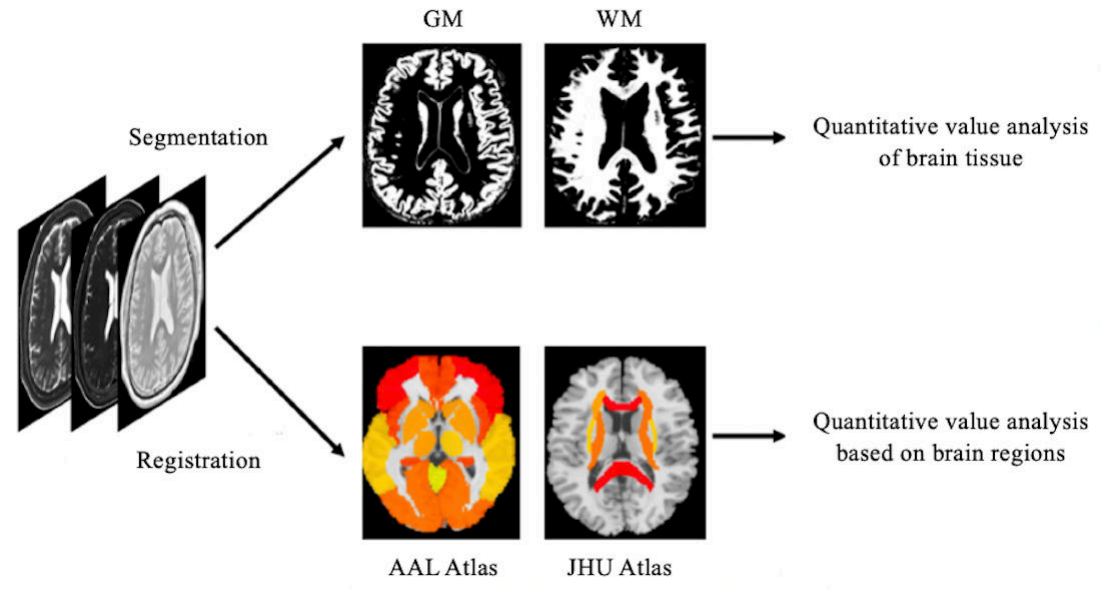
The process of quantitative value measurements for healthy participants. AAL: anatomical automatic labeling; GM: gray matter; JHU: Johns Hopkins University; WM: white matter.

The flowchart illustrating the process of quantitative value measurements is presented in Figure 2.

For healthy participants, quantitative value analysis of brain tissue and brain regions was conducted. First, brain tissue segmentation was performed on each individual’s quantitative map using the New Segment tool within SPM [20] software. Then, quantitative value extraction of gray matter (GM) and WM was performed using self-written programs in the MATLAB (The MathWorks Inc.) platform.

In addition to analyzing quantitative values for both GM and WM tissue, analysis was also performed by different brain regions for clinical evaluation and application. Using the anatomical automatic labeling atlas of the Montreal Neurological Institute (MNI), 10 GM regions of interest (ROIs) were created on anatomical structures, including frontal cortex, temporal cortex, parietal cortex, occipital cortex, insula, hippocampus, caudate nucleus, putamen, pallidum, and thalamus. Additionally, using the JHU-WhiteMatter-48 atlas, 9 WM ROIs were generated, including the middle cerebellar peduncle, genu of corpus callosum, splenium of corpus callosum, cerebral peduncle, internal capsule, anterior corona radiata, superior corona radiata, posterior corona radiata, and external capsule [21]. In all analyses, right and left quantitative data were averaged. The quantitative value of the corresponding brain region in the quantitative map reconstructed by DL and from routine scans was obtained for each individual for subsequent statistical analysis.

For the images of individuals with pathologies, an experienced radiologist used the ITK-SNAP software (version 3.8.0; [22]) to delineate the ROI of typical lesions manually and acquired the mean value of the signal in the ROI. To ensure paired comparison, identical ROI size and location were replicated on the DL reconstructed and reference routine scan images. A total of 12 ROIs were obtained from 7 (4.43%) patients (individual 3: 3 ROIs, individual 5: 3 ROIs, individual 7: 2 ROIs, and the remaining 4 individuals: 1 ROI each).

### Image Quality Evaluation

Two full-reference evaluation indices—peak signal-to-noise ratio (PSNR) and structural similarity image measure (SSIM)—were used in this study for image quality assessment [23]. The larger the value of the 2 indices, the better the image quality. The PSNR was defined by equation 5:


$$MSE=\frac{\sum_{m=1}^{M}\sum_{n=1}^{N}{\left[R\left(m,n\right)-I\left(m,n\right)\right]}^{2}}{M\times N}$$

(5) $$PSNR=20log\frac{L^{2}}{MSE}$$

The calculation method of SSIM is given by equation 6:

, (6) $$SSIM(X,Y)=\frac{\left(2\mu_{X}\mu_{Y}+C_{1})(2\sigma_{XY}+C_{2}\right)}{\left(\mu_{X}^{2}+\mu_{Y}^{2}+C_{1})(\sigma_{X}^{2}+\sigma_{Y}^{2}+C_{2}\right)}$$

$$C_{1}={\left(K_{1}L\right)}^{2},C_{2}={\left(K_{2}L\right)}^{2}$$

where μₓ and μᵧ, σₓ and σᵧ, and σₓᵧ are the local means, SDs, and cross-covariance for the DL quantitative images and routine scans, respectively, and $C_{1}$ and $C_{2}$ are 2 quantities used to stabilize the division in the case of a weak denominator, with L=65,535, $K_{1}$ = 0.01, and $K_{2}$= 0.03.

In addition, a no-reference image quality assessment method, the naturalness image quality evaluator (NIQE), was used to evaluate the quality of quantitative images reconstructed by DL, with fast scan and with routine scan, respectively [24]. Unlike PSNR and SSIM, the lower the value of NIQE, the less distortion and higher image quality.

### Statistical Analysis

For the quantitative analysis of brain tissue in healthy participants, Paired *t*-test was used to compare the differences in quantitative values between DL and routine scans, and equivalence was confirmed using two one-sided tests (TOST). Linear regression analysis and Bland-Altman analysis were used in the study, and we combined GM and WM to comprehensively evaluate overall bias. This method captures the overall trends and variability of different tissue types, which is crucial for evaluating the performance of DL reconstruction in clinically relevant contexts. For the region-based quantitative value analysis in healthy participants, coefficients of variation (CVs) were calculated within each imaging method (intragroup CV) and across each imaging method (intergroup CV). Intergroup CV was calculated using the average of the quantitative values from each method. For the images of individuals with pathologies, Paired *t*-test was used to compare quantitative values in different ROIs between groups. All statistical analyses were performed using SPSS 22.0 and GraphPad Prism 9. A *P* value <.05 was considered statistically significant.

### Ethical Considerations

This study was approved by the Medical Research Ethics Committees of Beijing Friendship Hospital, Capital Medical University (2020-P2-122-02). All participants provided informed consent before participating. Participants’ privacy and confidentiality were protected, with all data being deidentified during analysis. No compensation was provided for participation. No identifiable information of participants was included in the paper or supplementary materials in Multimedia Appendix 1. All procedures adhered to ethical guidelines for participant autonomy, safety, and confidentiality.

## Results

### Time Consumption

The average whole brain acquisition time was 4 minutes 55 seconds for routine scans, with an additional 1 minute for vendor postprocessing. Fast scans were completed in 1 minute 52 seconds; applying the SRGAN network trained in this study then reconstructed, the 512 by 512 quantitative T1/T2/PD maps were completed in approximately 1 second. Thus, the combined acquisition and reconstruction time was reduced from 5 minutes 55 seconds to 1 minute 53 seconds, promoting the wider clinical application of quantitative neuroimaging and synthetic MRI.

### Quantitative Value Accurac*y*

A paired *t* test showed significant differences in GM tissue values between DL and fast scans (T1: *P*<.001, PD: *P*=.002), while there was no significant difference in GM tissue values between DL and routine scans at T1 (*P*=.66) and PD (*P*=.18). However, compared with routine scans, T2 values still showed significant differences after DL (*P*<.001). For WM, compared with routine scans, there was no significant difference in T2 after DL (*P*=.11). Although there were still significant differences in T1 and PD, the differences were reduced compared with fast scans (Table 1).

**Table 1.** The quantitative value of brain tissue obtained by fast scan, deep learning (DL), and routine scan.

	Fast, mean (SD)	DL, mean (SD)	Routine, mean (SD)	*P* value^a^	*P* value^b^
GM^c^					
T1 (ms)	1200.03 (36.01)	1230.17 (34.12)	1232.65 (34.98)	<.001	.66
T2 (ms)	97.87 (2.07)	91.36 (2.60)	93.33 (3.50)	<.001	<.001
PD (%)	79.69 (2.35)	80.29 (1.51)	80.11 (1.23)	.002	.18
WM^d^					
T1 (ms)	814.88 (35.78)	855.06 (32.12)	837.86 (35.88)	<.001	<.001
T2 (ms)	84.57 (2.17)	80.23 (1.61)	79.92 (2.05)	<.001	.11
PD (%)	67.55 (1.94)	68.49 (1.44)	68.21 (1.29)	<.001	.01

a *t* test results between DL and fast scans.

b *t* test results between DL and routine scans.

c GM: gray matter.

d WM: white matter.

Setting the clinically acceptable brain tissue quantification value limit to ±5% of the mean values from the routine scans (T1_GM epsilon: 61.63 ms, T2_GM epsilon: 4.67 ms, PD_GM epsilon: 4.01%, T1_WM epsilon: 41.89 ms, T2_GM epsilon: 4.00 ms, and PD_WM epsilon: 3.41%), the 90% TOST CI for the brain tissue in 3 quantitative maps (T1_GM: −11.88 to 6.91, T2_GM: −2.48 to−1.46, PD_GM: −0.04 to 0.41, T1_WM: 10.94 to 23.46, T2_WM: −0.01 to 0.03, and PD_WM: 0.10 to 0.45) were all within the predefined margins (*P*<.001).

**Figure 3.**
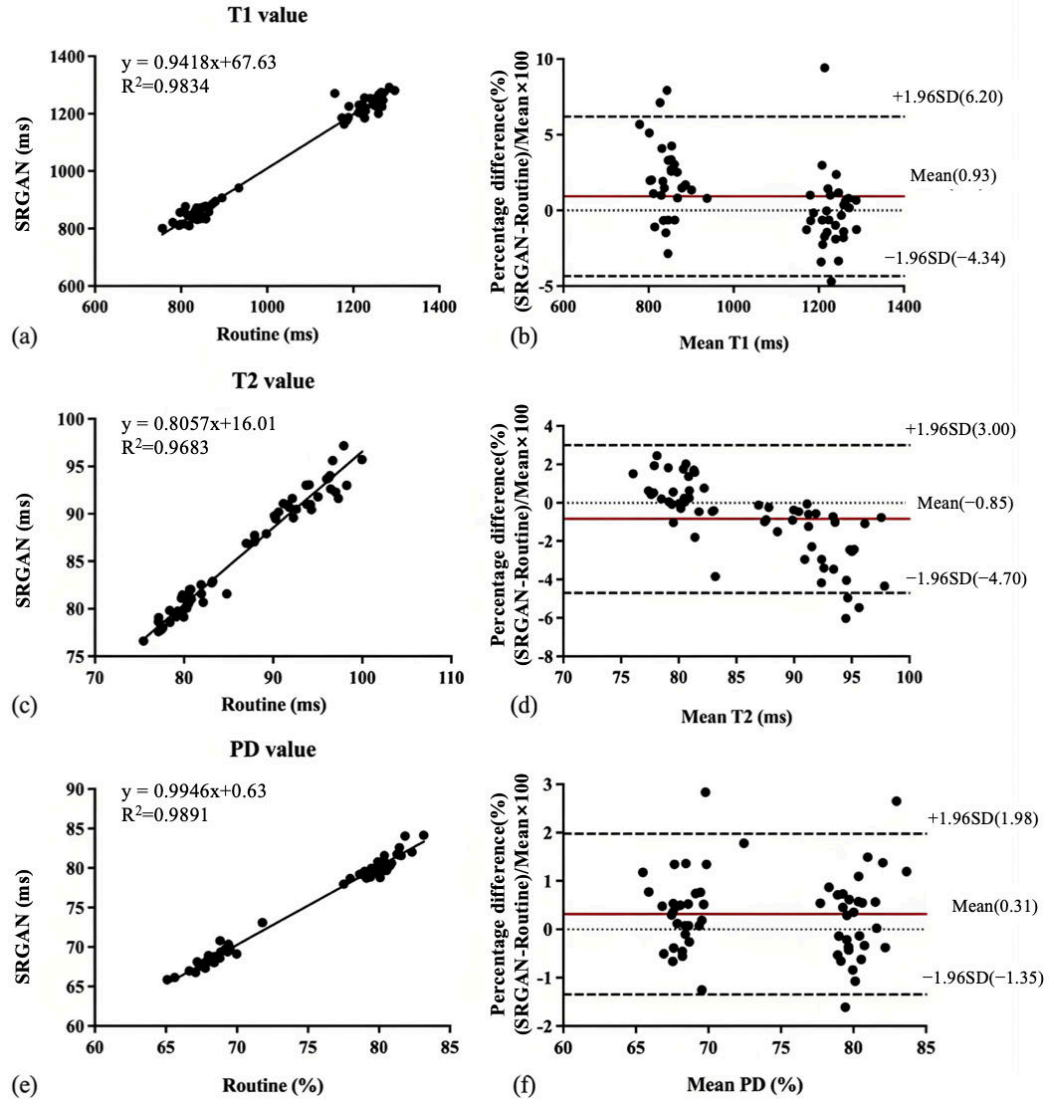
Scatterplots and Bland-Altman plot results for deep learning versus routine scans. The linear regression lines represent a strong linear relationship with a robust fit between the tissue value measurements of T1, T2, and proton density (PD) obtained from the 2 magnetic resonance imaging methods. Bland-Altman plots show mean percentage differences (solid red line) and the 95% CIs (dashed black line). SRGAN: superresolution generative adversarial network.

**Figure 4.**
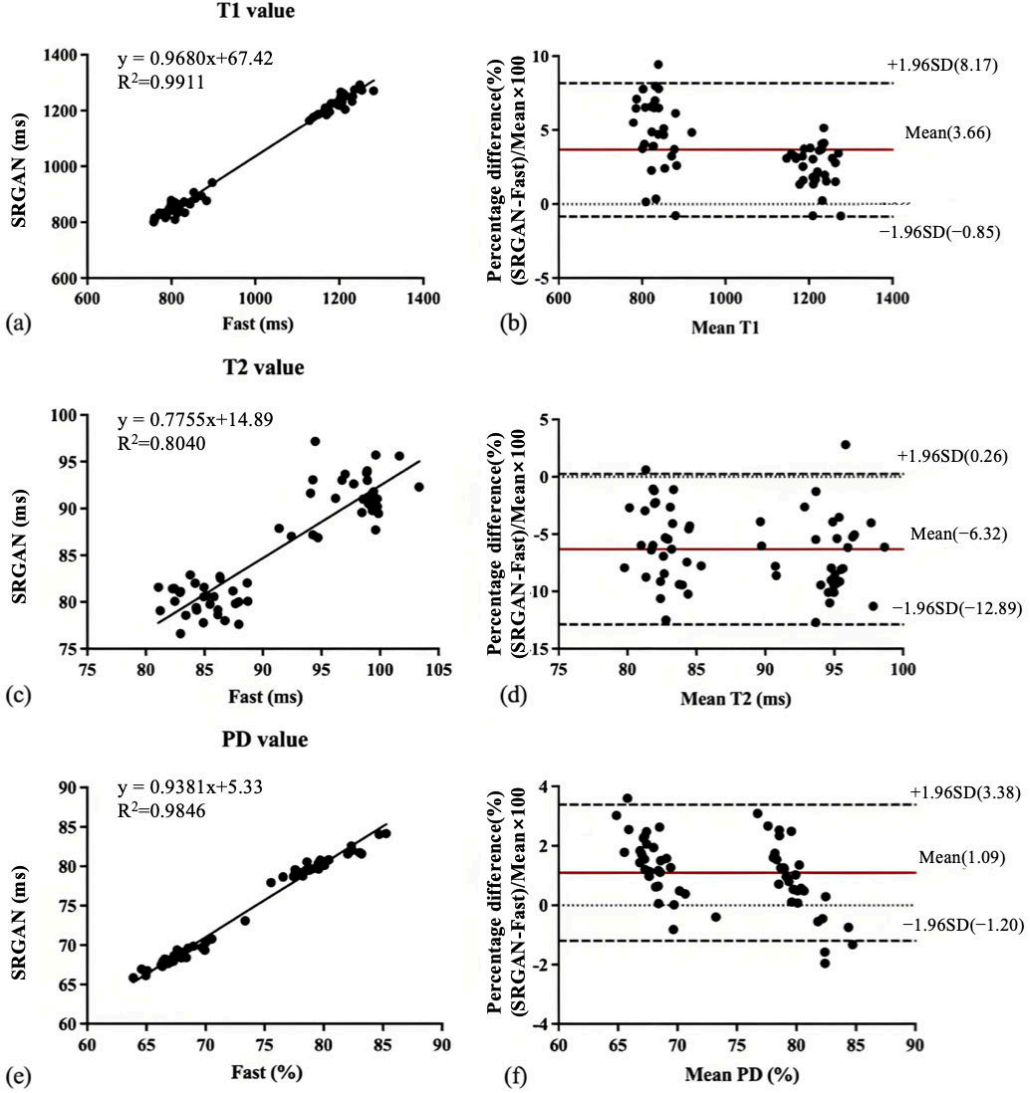
Scatterplots and Bland-Altman plot results for deep learning versus fast scans. The linear regression lines represent a strong linear relationship with a robust fit between the tissue value measurements of T1, T2, and proton density (PD) obtained from the 2 magnetic resonance imaging methods. Bland-Altman plots show mean percentage differences (solid red line) and the 95% CIs (dashed black line). SRGAN: superresolution generative adversarial network.

Figure 3 shows the relationship and Bland-Altman plots between the T1, T2, and PD values obtained by DL and routine scans (reference). The correlations to the reference value were excellent, as the *R*^2^ for the T1, T2, and PD values of DL compared to the reference value was 0.98, 0.97, and 0.99, respectively. The slopes of the linear regression were near 1.0 both for T1 (0.9418) and PD (0.9946). In contrast, the T2 values were moderate, as the slope of the linear regression was 0.8057 (Figure 3A,C,E). In addition, the average percentage bias for T1, T2, and PD was 0.93%, −0.85%, and 0.31%, respectively. The 95% limits of agreement were 6.20% to −4.34% for T1, 3.00% to −4.70% for T2, and 1.98% to −1.35% for PD (Figure 3B,D,F). Similarly, Figure 4 shows the relationship and Bland-Altman plots of the T1, T2, and PD acquired by DL and fast scans. The results of linear regression analysis also showed a strong correlation between the 2 methods in T1 and PD (T1: *R*^2^=0.99 and PD: *R*^2^=0.98), with a slightly lower correlation in T2 (*R*^2^=0.80). The slopes of the linear regression were near 1.0 both for T1 (0.9680) and PD (0.9381). In contrast, the T2 values were moderate, as the slope of the linear regression was 0.7755 (Figure 4A,C,E). The results of the bias trend of T1, T2, and PD between these 2 methods also showed similar results, that is, the mean percentage difference for T1 and PD was smaller (3.66% and 1.09%, respectively), whereas the mean percentage difference for T2 was slightly higher (−6.32%) (Figure 4B,D,F).

Table 2 presents the intragroup CV and intergroup CV of brain regions’ quantitative values acquired by DL and routine scans. The intragroup variability of the quantitative values obtained by these 2 methods is small, as the intragroup CV for the routine scan’s PD values ranges from 1.64% to 6.44%, and the PD values for DL range from 1.98% to 5.84%. The highest intragroup CV of T1 and T2 values was noted in the pallidum (T1: 18.77% vs 17.25%; and T2: 17.00% vs 16.33%). The intergroup CV was lower than the intragroup CV for all brain regions. For intergroup CV, there were no significant differences in the quantitative values of T1, T2, and PD for the WM region after DL reconstruction. However, significant differences in T2 values were observed in the GM regions, including the frontal cortex, temporal cortex, parietal cortex, occipital cortex, insula, and hippocampus.

Table 3 presents the quantitative values in ROI of typical lesions acquired by DL and routine scans. Compared with the quantitative values under routine scans, there were no significant differences in the quantitative values reconstructed by deep learning (T1, *P*=.67; T2, *P*=.73; and PD, *P*=.75).

**Table 2.** Intragroup and intergroup coefficient of variation (CV) for T1, T2, and proton density (PD) based on anatomical automatic labeling (AAL) and Johns Hopkins University (JHU) atlas in healthy individuals.

Brain region	Intragroup CV (%)			Intergroup CV (%) (T1/T2/PD)	*P* value (T1/T2/PD)
	DL^a^ (T1/T2/PD)	Routine (T1/T2/PD)			
AAL atlas					
Frontal	7.41/7.28/3.62	8.20/7.14/3.44		2.07/5.17/0.61	.29/.001/.94
Temporal	5.10/8.00/3.31	5.50/8.62/3.27		1.65/4.98/0.40	.38/.003/.83
Parietal	9.32/14.40/3.02	9.08/15.90/2.97		1.30/8.11/0.45	.38/<.001/.42
Occipital	7.43/11.07/2.15	7.19/13.32/2.41		1.72/5.10/0.47	.60/.005/.55
Insula	9.03/15.23/2.39	9.86/13.44/2.53		1.80/7.12/0.72	.95/.02/.36
Hippocampus	8.95/11.43/2.53	9.02/14.30/2.71		2.62/6.08/0.72	.22/.005/.34
Caudate	13.69/16.20/5.29	14.65/17.14/5.37		2.60/5.32/0.76	.70/.35/.92
Putamen	13.48/12.37/2.62	14.48/17.33/3.47		2.04/5.44/0.73	.99/.11/.92
Pallidum	18.77/16.33/2.26	17.25/17.00/2.61		3.54/3.37/0.85	.51/.56/.50
Thalamus	13.37/12.39/2.38	13.99/13.96/3.00		1.69/4.05/0.88	.77/.27/.67
JHU atlas					
MCP^b^	7.28/7.04/1.72	5.89/8.13/1.64		1.67/2.79/0.51	.50/.08/.28
GCA^c^	15.33/9.77/5.84	15.22/9.11/6.44		1.99/2.89/0.76	.87/.52/.55
SCA^d^	6.44/3.70/1.98	6.00/4.64/1.94		1.74/1.71/0.63	.19/.05/.13
CP^e^	10.16/7.81/4.77	9.62/7.54/5.01		2.73/2.55/1.08	.41/.14/.55
IC^f^	9.55/4.29/3.95	9.63/4.52/4.20		1.30/1.74/0.68	.83/.21/.45
ACR^g^	9.01/5.62/2.94	9.78/6.91/3.86		1.97/1.34/0.91	.92/.97/.98
SCR^h^	9.95/4.68/3.73	8.35/4.95/3.07		2.16/1.31/1.03	.27/.14/.28
PCR^i^	14.19/3.72/4.25	12.20/4.59/3.69		2.60/2.49/0.96	.42/.03/.23
EC^j^	11.32/5.02/2.82	11.23/4.89/3.36		1.38/0.92/0.95	.65/.69/.21

a DL: deep learning.

b MCP: middle cerebellar peduncle.

c GCA: genu of corpus callosum.

d SCA: splenium of corpus callosum.

e CP: cerebral peduncle.

f IC: internal capsule.

g ACR: anterior corona radiata.

h SCR: superior corona radiata.

i PCR: posterior corona radiata.

j EC: external capsule.

**Table 3.** Quantitative value in the region of interest (ROI) of typical lesions acquired by deep learning (DL) and routine scan.

ROI	Description	T1^a^ (ms)		T2^b^ (ms)		PD^c^^,^^d^ (%)	
		DL	Routine	DL	Routine	DL	Routine
1	WMH^e^	1019.68	1052.20	100.84	112.9	80.70	79.16
2	WMH	1034.41	1052.78	96.32	96.71	75.21	77.09
3	WMH	955.68	1013.20	94.78	104.52	70.77	72.97
4	CI^f^	1217.30	1398.16	126.53	149.71	80.00	81.26
5	Encephalomalacia	3597.26	3683.45	310.67	276.99	102.83	106.59
6	WMH	1088.41	1214.51	109.50	121.23	82.11	84.70
7	CI	1832.00	1679.00	136.10	133.80	90.50	90.90
8	Encephalomalacia	3719.04	3892.54	292.16	272.32	101.31	104.98
9	WMH	1734.53	1517.03	163.45	182.01	85.14	86.37
10	WMH	1171.00	1238.00	110.60	124.70	80.70	81.10
11	Encephalomalacia	3666.00	3469.00	350.20	295.80	107.80	106.30
12	WMH	1253.00	1285.00	113.20	105.20	78.70	83.00

a *P*=.67.

b *P*=.73.

c PD: proton density.

d *P*=.75.

e WMH: white matter hyperintensities.

f CI: cerebral infarcts.

### Image Quality

**Figure 5.**
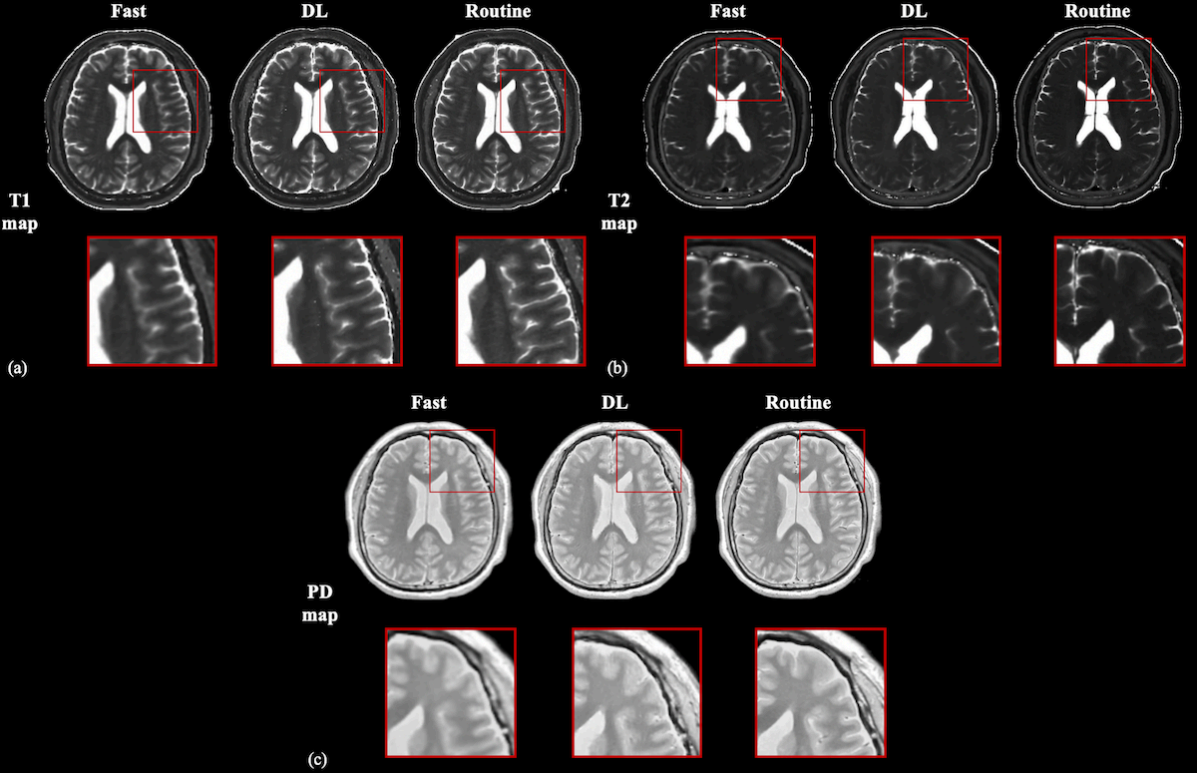
Representative results of the proposed method. Results of fast scans (left), deep learning (DL) reconstruction (middle), and routine scans (right) are displayed for T1 map, T2 map, and proton density (PD) map.

Figure 5 and Tables4  5 compare image quality among the 3 protocols. After SRGAN reconstruction, DL maps simultaneously preserved quantitative accuracy and outperformed fast acquisitions in every objective metric, demonstrating high image quality.

**Table 4.** Comparison of image quality between the deep learning quantitative maps and routine scans using the structural similarity image measure (SSIM) and peak signal-to-noise ratio (PSNR).

	PSNR, mean (SD)	SSIM, mean (SD)
T1 maps	21.94 (2.36)	0.82 (0.08)
T2 maps	29.14 (1.81)	0.93 (0.03)
PD^a^ maps	58.01 (3.15)	0.99 (0.01)

a PD: proton density.

**Table 5.** The naturalness image quality evaluator of quantitative maps under fast scan, deep learning (DL), and routine scan.

	Fast scan, mean (SD)	DL, mean (SD)	Routine, mean (SD)
T1 maps	29.46 (6.42)	13.36 (1.88)	10.90 (1.84)
T2 maps	30.56 (5.57)	18.79 (1.46)	15.21 (1.78)
PD^a^ maps	34.66 (2.99)	11.74 (2.17)	10.85 (2.53)

a PD: proton density.

## Discussion

### Principal Findings

In this paper, we trained an SRGAN network to reconstruct whole brain T1, T2, and PD maps from accelerated synthetic MRI data. Experimental results show that the application of DL for reconstruction can generate quantitative maps from fast scans with a 50% reduction in acquisition time. The quantitative instability caused by LR acquisition is reduced, with no significant difference between the reconstructed and routine scan values, and intra- and inter-CVs are small in most brain regions. Our study also found that the resulting maps still allow clear visual detection of common pathologies, thereby helping to shorten clinical scanning time.

### Comparison With Prior Work

In previous studies, the use of commercial algorithms for fast image reconstruction resulted in a total brain acquisition time exceeding 3 minutes [25]. Our research further reduces the collection time to less than 2 minutes while maintaining accuracy. DICOM raw data were used as input and output for the network, which conforms to the standard practice of DICOM image processing, where metadata (including rescaled slopes) are crucial for accurate image analysis and quantitative measurement. By adhering to these specifications, the study maintains the integrity of the qMRI data, facilitating a reliable evaluation of the DL reconstruction techniques’ impact on the accuracy of quantitative brain tissue values.

The MDME sequence was used to acquire the quantitative maps, and several studies have investigated the repeatability and reproducibility of quantitative values using this sequence. In this study, we applied multiple quantitative metrics and statistical methods to analyze differences among DL reconstructed, fast scan, and routine scan values. The results of our study, as indicated by the paired *t* tests, provide valuable insights into the performance of DL reconstruction and demonstrate that it improves the accuracy of fast quantitative MRI scans, bringing tissue-specific T1, T2, and PD values closer to those of routine clinical acquisitions. In GM, the method achieved no statistically significant difference compared to routine scans. However, a small but statistically significant underestimation in T2 remained. A previous study examining the influence of parameter modifications on MDME-derived quantitative values reported that alterations in acquisition matrix or acceleration factor induced negligible variance in measured constants, with the exception of CSF T2 estimation [26,27]. Notably, this investigation was performed on an isolated, homogeneous phantom; consequently, partial volume effects and other anatomical confounders were absent. The present fast scan protocol, in contrast, simultaneously varies both matrix size and acceleration factor without a commensurate increase in the number of echo times. This divergence in experimental design may compromise the accuracy of T2 mapping—particularly through reduced sampling density in echo space—thereby introducing systematic bias into the fitted relaxation constants. However, TOST analysis further indicates that the 90% CIs of all brain tissue parameters in the 3 quantitative maps are within clinically acceptable ranges, providing the possibility for the clinical applications of DL quantitative reconstruction.

In our quantitative analysis, the linear regression slope for T2 values (DL vs routine) was 0.8057, which deviates from the ideal value of 1.0. Clinically, this may affect the diagnostic accuracy of high T2 lesions, leading to misdiagnosis or delayed intervention. Most of the samples in this study were healthy volunteers, and the accuracy of DL for T2 values under specific pathological manifestations was not validated. Such bias may arise primarily from the L1 loss component, which enforces pixel-wise fidelity at the expense of high-frequency details and can lead to oversmoothing—particularly in regions with high tissue contrast or rapid T2 decay. Consequently, although the average T2 error across healthy tissue may satisfy TOST criteria, it may reduce the accuracy of this method in extreme cases of dynamic range that often occur in pathology. Despite this limitation, the SRGAN model demonstrated good performance in overall image quality metrics, indicating its practicality in routine clinical workflows, although it requires appropriate attention.

Linear regression and Bland-Altman plots also demonstrated that the vast majority of quantitative parameters exhibited high correlation and agreement between the DL and routine scans, with 95% of data points lying within the limits of agreement, which is in line with previous research [28,29]. In contrast, T2 estimates derived from the DL exhibited a marked bias relative to the reference (Figure 3 vs Figure 4). These findings indicate that the DL model successfully compensates for systematic errors introduced by the fast scan, yielding quantitative values that closely approximate those obtained under routine scans. Such error mitigation directly supports the primary objective of this study: to establish the feasibility of using DL for accurate, ultrafast quantitative MR reconstruction.

In addition to measuring the quantitative values of gray and WM, the study also applied classic atlases from brain science analysis: the anatomical automatic labeling and the Johns Hopkins University atlases [30]. These atlases divide the brain into regions based on structure or function, which allows for a more comprehensive characterization of the accuracy and reliability of the quantitative values. Generally, the intragroup CV should ideally be below 10%, and the intergroup CV should remain under 15%, yet a significant intragroup CV was observed in deep GM nucleus regions, such as pallidum. This may be due to the inhomogeneity of B1 and differences in data acquisition parameters or variations in the age and gender of the individuals, which have also been shown and analyzed in previous studies [31-33]. Additionally, these numerical disparities may stem from spatial registration errors between individual quantitative maps and brain region templates, potentially affecting the extraction of quantitative values. Our study also observed significant differences in the T2 CVs between DL and routine scans in some GM brain regions. This difference indicates that although the SRGAN model has good consistency at the overall organizational level, it may introduce variability at the regional scale, which may be due to the model’s sensitivity to local texture or noise, potentially limiting its practicality in precise quantitative neuroimaging applications. However, the intra- and inter-group CV for the remaining brain regions remained below 5%, especially for PD values, which show minimal intragroup and intergroup variation across all brain regions. This implies that T2 is relatively sensitive in quantitative analysis and should be a key focus in disease analysis.

The outlined lesion areas in the study are predominantly distributed in the WM. The average T1, T2, and PD quantitative values within these lesion areas exceed 1000 milliseconds, 100 milliseconds, and 80 milliseconds, respectively, which are higher than the ranges observed in the healthy participants’ quantitative values. Furthermore, different types of lesions exhibit distinct quantitative values. WM hyperintensities and acute-phase cerebral infarction have similar quantitative values. In contrast, the quantitative values in encephalomalacia are significantly higher than those in the other 2 types of lesions, particularly the T1 values within encephalomalacia (approaching 4000 ms), which are similar to the T1 value of cerebrospinal fluid because the components of encephalomalacia are similar to cerebrospinal fluid. These findings align with clinical knowledge, indicating that lesion areas generally have longer T1 and T2 relaxation times. Importantly, the quantitative values obtained through DL still maintain diagnostic efficacy. Through careful examination of quantitative values, it was found that with a small amount of lesion data, although the difference in quantitative values between DL and routine scans did not reach statistical significance, 5 reconstructed T2 values were lower than those of the routine scan in 7 WMH and infarct ROIs. Given that these lesions have already exhibited high T2 relaxation times (usually >100 ms), any systematic underestimation may weaken their abnormal manifestations. This may be because superresolution networks are only trained on quantitative images of healthy adults and do not include any pathological cases. Therefore, the model has not yet learned the characteristic diastolic curves of common brain injuries, such as significant prolongation of WM hyperintensities on T2. This lack of exposure may lead to the network interpreting high T2 values in lesions as outliers or reconstruction artifacts, thereby regressing these values to the healthy tissue distribution. Therefore, the sample size will continue to be expanded in the future to verify the quantitative accuracy of DL reconstruction in various neurological diseases.

While the DL maps have basically met the predefined quantitative equivalence margins, the no-reference NIQE metric reveals a residual perceptual gap: compared with fast scanning, the DL image quality has significantly improved (as indicated by a decreased NIQE score), but compared with routine scans, the NIQE scores of the 3-parameter images are slightly higher. This suggests that the current model ensures the accuracy of clinical quantification and significantly improves accuracy on the basis of fast scans, but further improvement is needed to achieve complete perceptual equality with routine HR imaging.

### Limitations

This study still has some limitations. First, the study adopted the widely validated SRGAN framework and focused on quantitative agreement and image quality comparison; however, emerging architectures such as ESRGAN or SwinIR were not compared, nor were traditional non-DL upsampling methods used as baselines. Second, the network was trained exclusively on data acquired with a GE 3.0 T scanner and entirely on normal-appearing brain tissue (only 7 patients with 3 common lesion types were used for testing), so the performance of the method on pathological tissue is a preliminary proof of concept rather than evidence of clinical equivalence. The small number of pathology cases limits statistical power and generalizability to diverse or high-contrast lesions, which may pose a barrier to routine clinical deployment. Third, based on the current amount of data, using the training set and testing set with a single subject-level split of 8:2 may prevent data leakage from related slices but may not fully capture the variability between multiple random partitions. Due to limited training data, the model runs on 2D slices instead of 3D volumes, which may result in a loss of spatial continuity between slices. Fourth, acceleration was limited to an *R* of 3, reconstruction was performed on postprocessed quantitative maps, raw k-space data were not used, and weighted images generated from the quantitative maps were not evaluated. Additionally, the ground truth images have been reconstructed from a matrix of 320 by 256 to a matrix of 512 by 512, and thus, the model learns to reproduce the interpolated image content rather than recovering genuine high-frequency details that would be present in a natively HR acquisition. Finally, evaluation was performed based on quantitative metrics without introducing a qualitative visual assessment by radiologists.

### Future Directions

Future research should focus on the following improvements: systematically compare the current SRGAN implementation with newer superresolution networks and quantify the individual contributions of perceptual and adversarial losses to quantitative accuracy, and compare them with non-DL baseline reconstruction methods to better illustrate the benefits of DL. A prospective cohort including demyelinating disease, tumor edema, and microhemorrhages will be enrolled, and the model will be fine-tuned or retrained if necessary before wider clinical deployment. To improve robustness, we will collect both k-space and image-domain data and develop a dual-domain joint reconstruction framework. Future work will explore 3D or slice-aware architectures based on richer training data and computing resources and attempt to use different data partitioning methods to validate generalization further. Finally, the weighted images derived from the superresolved quantitative maps will be generated and assessed for diagnostic quality.

### Conclusions

In conclusion, accelerating synthetic MRI is pivotal for its broader clinical adoption in neuroimaging. In this study, we undertook the optimization and enhancement of images acquired through fast synthetic MRI via DL-based reconstruction techniques. Our efforts may result in a remarkable 50% reduction in clinical scan time, all the while achieving better image quality. Importantly, reconstructed T1 and PD maps show excellent agreement with reference scans, supporting their potential for reliable quantification, although T2 values exhibit a consistent underestimation at higher intensities. Despite this limitation, the overall strong correlations and low average biases suggest that using DL methods to mitigate biases in quantitative values is feasible. Furthermore, the shortened scan time not only enhances patient comfort but also reduces the likelihood of motion artifacts. This optimization streamlines the scanning workflow, minimizes redundancy, and maximizes the efficient use of MRI machines.

## Supplementary material

10.2196/79389 Multimedia Appendix 1 The details of network structure and loss function.

## Abbreviations

AAL: anatomical automatic labeling
CV: coefficient of variation
DL: deep learning
GM: gray matter
HR: high resolution
LR: low resolution
MRI: magnetic resonance imaging
NIQE: naturalness image quality evaluator
PD: proton density
PSNR: peak signal-to-noise ratio
ReLU: rectified linear unit
ROI: region of interest
SRGAN: superresolution generative adversarial network
SSIM: structural similarity image measure
TOST: two 1-sided tests
WM: white matter

## References

[1] Cashmore MT, McCann AJ, Wastling SJ, McGrath C, Thornton J, Hall MG (2021). Clinical quantitative MRI and the need for metrology. Br J Radiol.

[2] Warntjes JBM, Dahlqvist O, Lundberg P (2007). Novel method for rapid, simultaneous T1, T2*, and proton density quantification. Magn Reson Med.

[3] Blystad I, Warntjes JBM, Smedby O, Landtblom AM, Lundberg P, Larsson EM (2012). Synthetic MRI of the brain in a clinical setting. Acta Radiol.

[4] Gao W, Yang Q, Li X (2022). Synthetic MRI with quantitative mappings for identifying receptor status, proliferation rate, and molecular subtypes of breast cancer. Eur J Radiol.

[5] Zhang Z, Li S, Wang W (2023). Synthetic MRI for the quantitative and morphologic assessment of head and neck tumors: a preliminary study. Dentomaxillofac Radiol.

[6] Ma L, Lian S, Liu H (2022). Diagnostic performance of synthetic magnetic resonance imaging in the prognostic evaluation of rectal cancer. Quant Imaging Med Surg.

[7] Li M, Fu W, Ouyang L (2023). Potential clinical feasibility of synthetic MRI in bladder tumors: a comparative study with conventional MRI. Quant Imaging Med Surg.

[8] Bauer S, Markl M, Honal M, Jung BA (2011). The effect of reconstruction and acquisition parameters for GRAPPA-based parallel imaging on the image quality. Magn Reson Med.

[9] Goodfellow I, Pouget-Abadie J, Mirza M (2020). Generative adversarial networks. Commun ACM.

[10] Mardani M, Gong E, Cheng JY (2019). Deep generative adversarial neural networks for compressive sensing MRI. IEEE Trans Med Imaging.

[11] Quan TM, Nguyen-Duc T, Jeong WK (2018). Compressed sensing MRI reconstruction using a generative adversarial network with a cyclic loss. IEEE Trans Med Imaging.

[12] Yang G, Yu S, Dong H (2018). DAGAN: deep de-aliasing generative adversarial networks for fast compressed sensing MRI reconstruction. IEEE Trans Med Imaging.

[13] Liu Y, Liu Y, Vanguri R (2021). 3D isotropic super-resolution prostate MRI using generative adversarial networks and unpaired multiplane slices. J Digit Imaging.

[14] Su PY, Shih HJ, Xu JL (2025). Rapid liver fibrosis evaluation using the UNet-ResNet50-32 × 4d model in magnetic resonance elastography: retrospective study. JMIR Med Inform.

[15] Ran M, Hu J, Chen Y (2019). Denoising of 3D magnetic resonance images using a residual encoder-decoder Wasserstein generative adversarial network. Med Image Anal.

[16] Latif S, Asim M, Usman M, Qadir J, Rana R (2018). Automating motion correction in multishot MRI using generative adversarial networks. arXiv.

[17] Qiu S, Chen Y, Ma S (2022). Multiparametric mapping in the brain from conventional contrast-weighted images using deep learning. Magn Reson Med.

[18] Liu Y, Niu H, Ren P (2022). Generation of quantification maps and weighted images from synthetic magnetic resonance imaging using deep learning network. Phys Med Biol.

[19] Don H, Supratak A, Mai L Tensorlayer: a versatile library for efficient deep learning development.

[20] SPM8. UCL: Department of Imaging Neuroscience.

[21] Zheng Z, Liu Y, Yin H (2024). Evaluating T1, T2 relaxation, and proton density in normal brain using synthetic MRI with fast imaging protocol. Magn Reson Med Sci.

[22] itk-SNAP.

[23] Wang Z, Bovik AC, Sheikh HR, Simoncelli EP (2004). Image quality assessment: from error visibility to structural similarity. IEEE Trans Image Process.

[24] Saad MA, Bovik AC, Charrier C (2012). Blind image quality assessment: a natural scene statistics approach in the DCT domain. IEEE Trans on Image Process.

[25] Kim E, Cho HH, Cho SH (2022). Accelerated synthetic MRI with deep learning-based reconstruction for pediatric neuroimaging. AJNR Am J Neuroradiol.

[26] Zheng Z, Yang J, Zhang D (2022). The effect of scan parameters on T1, T2 relaxation times measured with multi-dynamic multi-echo sequence: a phantom study. Phys Eng Sci Med.

[27] Hirano Y, Ishizaka K, Sugimori H (2024). Assessment of accuracy and repeatability of quantitative parameter mapping in MRI. Radiol Phys Technol.

[28] McAllister A, Leach J, West H, Jones B, Zhang B, Serai S (2017). Quantitative synthetic MRI in children: normative intracranial tissue segmentation values during development. AJNR Am J Neuroradiol.

[29] Saccenti L, Andica C, Hagiwara A (2019). Brain tissue and myelin volumetric analysis in multiple sclerosis at 3T MRI with various in-plane resolutions using synthetic MRI. Neuroradiology.

[30] Rolls ET, Huang CC, Lin CP, Feng J, Joliot M (2020). Automated anatomical labelling atlas 3. Neuroimage.

[31] Hagiwara A, Fujimoto K, Kamagata K (2021). Age-related changes in relaxation times, proton density, myelin, and tissue volumes in adult brain analyzed by 2-dimensional quantitative synthetic magnetic resonance imaging. Invest Radiol.

[32] Hagiwara A, Hori M, Cohen-Adad J (2019). Linearity, bias, intrascanner repeatability, and interscanner reproducibility of quantitative multidynamic multiecho sequence for rapid simultaneous relaxometry at 3 T: a validation study with a standardized phantom and healthy controls. Invest Radiol.

[33] Zheng Z, Liu Y, Wang Z, Yin H, Zhang D, Yang J (2024). Evaluating age-and gender-related changes in brain volumes in normal adult using synthetic magnetic resonance imaging. Brain Behav.

