# Supplementary material for: Two-Minute Deep Learning–Powered Brain Quantitative Mapping: Accelerating Clinical Imaging With Synthetic Magnetic Resonance Imaging
Source: JMIR Med Inform. 2026 Jan 23;14:e79389. doi: 10.2196/79389 (PMC12833913; doi:10.2196/79389)
Supplement: Multimedia Appendix 1 [file medinform-v14-e79389-s001.docx]

*Network Architecture*

The detailed generator network structure is shown in Figure 1(a). It starts with a 9 x 9 convolutional filter and connects 5 residual blocks, each of which consists of two 3 x 3 convolutional layers alternating with batch normalization layers (BN), and activation is performed using a rectified linear unit (ReLU) function. After the residual network, a 3 x 3 convolution and a 1 x 1 convolution are connected, and a subpixel convolutional layer is used to up-sample the image. In the generator network, except for the last layer that uses tanh as the activation function, all other layers use ReLU as the activation function. The discriminator network structure is shown in Figure 1(b). In the discriminator network D, our model first uses a structural form of eight 3 x 3 convolutional layers and Leaky ReLU function activation. All convolutional layers except the first have batch normalization layers. The Sigmoid activation function is applied to the last fully connected layer, the probability of discriminating whether the input high-resolution image is a real high-resolution image or an image generated by the generator. The network architecture includes a pre-trained VGG-19 network, which is used for feature extraction and loss function calculation.

*Loss Function*

The pixel-by-pixel error method used is the L1 loss function, also known as the mean absolute error (MAE), which is calculated by equation (1). Although SRGAN completely discards the pixel-by-pixel error, we still add this error in a certain proportion during the actual training process to amplify the difference and guide the optimization of the model. The cross-entropy loss function of the discriminator is used as the adversarial error of the network, and the calculation method is expressed by equation (2). In addition to using the adversarial error, SRGAN also uses a content error, which is defined as the Euclidean distance between the super-resolved image and the feature map of the reference image, and the calculation method is expressed by formula (3). The content error is used to align the content of low-resolution images and high-resolution images, which plays the same role as the mean square error. In this network, the pre-trained VGG-19 network is used to extract the feature parameter map. Therefore, the loss function in this network is described by equation (4).

| $L_{mae}=\frac{1}{\mathrm{CHW}}\sum_{i, j,k} \vert I_{i,j,k}^{DL}-I_{i,j,k}^{GT}\vert$ | (1) |
| --- | --- |

where, $I_{DL}$is the output image of the generator, and $I_{GT}$is the input reference image.

| $L_{gan}=-\sum_{i} logD(G\left( I_{DL} \right))$ | (2) |
| --- | --- |

where, G and D is the generative network and the discriminative network, respectively.

| $L_{VGG}=\frac{1}{C_{j}H_{j}W_{j}}\sum_{x=1}^{W_{j}} \sum_{y=1}^{H_{j}} \sum_{c=1}^{C_{j}} {(\emptyset_{j}({G\left( I_{DL} \right)}_{x,y,c})-\emptyset_{j}({G\left( I_{GT} \right)}_{x,y,c}))}^{2}$ | (3) |
| --- | --- |

where, ${(\emptyset}_{j}$) is the feature map obtained from the jth convolutional layer of the VGG-19 network, $C_{j}$, $H_{j}$and $W_{j}$ are the number of channels, height and width of the feature map, $I_{DL}$is the output image of the generator, $I_{GT}$is the input reference image.

| $L_{total}=L_{mae}+{10}^{-3}L_{gan}+{2*10}^{-6}L_{VGG}$ | (4) |
| --- | --- |
